# On the slope of the regression between stem cell divisions and cancer risk, and the lack of correlation between stem cell divisions and environmental factors-associated cancer risk

**DOI:** 10.1371/journal.pone.0175535

**Published:** 2017-05-16

**Authors:** Cristian Tomasetti, Bert Vogelstein

**Affiliations:** 1 Division of Biostatistics and Bioinformatics, Johns Hopkins Kimmel Cancer Center, Baltimore, Maryland, United States of America; 2 Department of Biostatistics, Johns Hopkins Bloomberg School of Public Health, Baltimore, Maryland, United States of America; 3 Ludwig Center & Howard Hughes Medical Institute, Johns Hopkins Kimmel Cancer Center, Baltimore, Maryland, United States of America; West Virginia University, UNITED STATES

We are pleased that the analyses on smoking and radiation performed by Little et al. support the idea that the effects of various environmental factors on cancer risk do not correlate significantly with the lifetime number of stem cell divisions. This provides further evidence that the correlation we obtained for the U.S., and similarly found across the rest of the world, is in general not due to environmental factors but rather is due to intrinsic ones. We also point out mathematical and conceptual mistakes in the analysis of Little et al. that led them to erroneous conclusions.

## Introduction

We are delighted that many investigators are considering the issues raised by our previous study. The recent article by Little et al. highlights some of these important issues and gives us an opportunity to comment on them.

## Results and discussion

Little et al. [1] misrepresent our findings [2] by making incorrect statements that are similar to those previously found in some media. The problem with these types of statements has been already pointed out by others [3]. Specifically, the authors at times confuse the concept of absolute risk with that of relative risk. We never suggested that “most cancers arose by chance”—as stated in the first sentence of the Abstract of [1]. Instead, we stated that the variation in cancer risk is largely explained by stem cell divisions [2]. We interpreted this correlation as indicating that replicative mutations associated with stem cell divisions (bad luck) was largely responsible for this variation. The difference between absolute risk and relative risk is fundamental in epidemiological statistics. In fact, the first word in the title of our paper is "Variation". While some journalists caused confusion by not differentiating between these two very different concepts, we would hope that this fundamental mistake is not perpetuated in the scientific literature. A similar mistake is reiterated in the Discussion section of Little et al., where it is stated that we incorrectly “infer that most cancers are just due to bad luck”. No such inferences were made by us, as we were well aware of the differences between absolute and relative risks.

In their new analysis, Little et al. assumed a modified version of the standard multistage carcinogenesis model of Armitage and Doll [4]. The authors attempt to justify the choice of an Armitage-Doll model by stating that “much use has been made” (p. 2) of this model in the past. The same justification could be made if they were to use aether models to explain the transmission of electromagnetic and gravitational forces. Neither the aether model nor the original Armitage and Doll model are now considered appropriate for reasons that became apparent only after these models were posited. The Armitage and Doll model does not account for clonal expansions, the fundamental component of carcinogenesis. A related error lies in the statement that “the Armitage-Doll model has also been used to determine the number of driver-gene mutations associated with two types of cancer” (p. 2), referring to another of our recent papers [5]. This statement is incorrect, as we used a different mathematical model, partially based on that of Durrett et al. [6], to arrive at the conclusions in [5].

Little et al. attempted to incorporate clonal expansions into their analysis (their equations 2 to 7), stating that their equations are as “has been done by Tomasetti et al. [5], based on approximations of Durrett and Moseley [6]”. This is also incorrect, as their equations do not follow from the mathematical principles described in [5], [6]. Little et al. used a different model and did not provide a justification for it. Thus, the Little et al. claim that they use “the same modified Armitage-Doll model” that we used is also incorrect. We did not use an Armitage-Doll type model, or any modified version of it. Moreover, the model used by Little et al. did not include the developmental phase of tissues. While in some tissues this will not affect the results substantially, in others it is critical because the great majority of cell divisions within those tissues occurs during their development phases.

**Fig 1:**
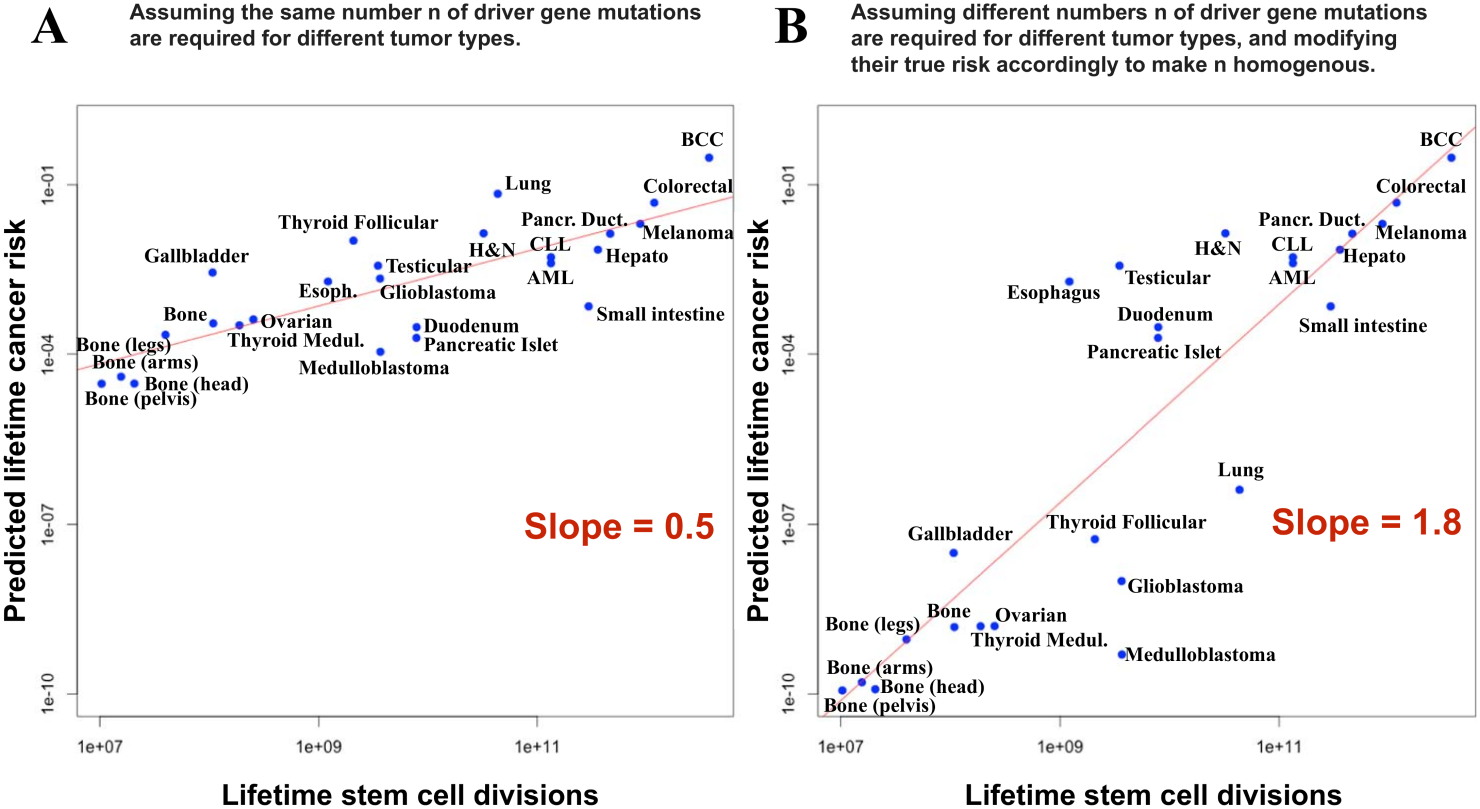
Dependence of the slope on the degree of heterogeneity in the number of required driver genes across tissues. (A) Regression line (red), for predicted cancer risk if it is assumed that all cancer types require exactly the same number of driver gene mutations, n, as assumed by Little et al. [1]. The slope of the regression line is 0.5. (B) Regression line (red), for predicted cancer risk if it is assumed that different cancer types require different numbers n of driver gene mutations (two in bone, ovarian, thyroid, gallbladder, brain, and lung cancer, and three drivers in all other cancer types.) To make the data points homogeneous in n, in order to regress on them, their risk was modified according to an Armitage and Doll model, for simplicity, with a mutation rate u = 5x10^-7^ per gene per cell division. All other variables were identical to those in A. The slope of the regression line is 1.8.

Little et al. also claim that the fact that the slope of the regression line relating stem cell divisions to cancer incidence is less than 1.0 –something already observed by us [7]—provides evidence against our conclusions. Let’s review their approach: based on the flawed mathematical model described above and making the strong (as admitted by Little et al.) assumption that all cancers types require the same number of driver genes to be mutated, Little et al. estimate that the slopes should be >3, and not the observed <1. The first problem is that the slopes observed in cancer incidence data are not constant, as assumed by Little et al., but actually non-linear (for one of many examples, see Figure 2.3 of [8]). Second, the number of driver genes that must be mutated is clearly not constant across cancers, as mistakenly assumed by the authors. Persuasive evidence about this point is provided by considering the distribution of driver mutations found across different cancer types, and across patients, as shown in Fig 5 of [9]. Thus, the fact that the slope of their regressions is < 1 is not a novel finding and more importantly does not provide evidence for or against our conclusions. And performing the analysis by leaving out leukemia, bone and thyroid cancers, and again assuming all other cancers require the same number of driver gene mutations, will not fix the problem. As pointed out in [7], a slope <1 can be easily explained by simple heterogeneity in the number of driver mutations across cancer types. If the theoretical cancer incidence, based on modeling, is plotted against the number of stem cell divisions, then the slope of the regression can vary markedly depending simply on the assumed heterogeneity in the number of driver genes. An example is shown in Fig 1. Such plots should not be confused with the observed (rather than modeled) cancer incidence plots in [1].

The Extra Risk Score (ERS), and related RBERS, were the first measures to assess the evidence of the role played by environmental and inherited factors in particular cancer types. As consistent with the goals of [2], these measures can reflect only relative risk with respect to other cancers, not absolute risk. A cancer with a higher ERS is evidence of a larger role of those factors in that cancer type than in the other cancers [2]. Interestingly, the authors use radiation and smoking data to test our ERS. Such tests are unjustified. Why should a tissue with a higher ERS than another tissue be more affected by one single environmental factor? For example, liver cancers have high ERS, but this doesn't mean that smoking or radiation plays a larger role in liver cancers than in other cancers. The lack of correlation between ERS and radiation- or smoking-induced cancer risk is expected from the data published in [2].

On the other hand, the lack of correlation found by Little et al. [1] is a relevant and informative result. It provides provide further evidence that substantially strengthens our preliminary analyses on the effects of atomic radiation [7, 10] and on the effects of stem cell numbers on the proportion of cancer cases explained by environmental factors [10]. In these studies, we showed a lack of correlation between the effects of the atomic bomb on cancer risk and the number of stem cell divisions in that tissue. Given that radiation and smoking are the strongest and most pervasive environmental carcinogens known today, the results of Little et al. provide important, independent evidence that environmental factors do not substantially contribute to the correlation discovered in [2].

## Conclusions

There is also an important philosophical point that the paper by Little et al., and our response to it, brings up. The main objective of our paper [2] was to document a correlation between the lifetime number of stem cell divisions and cancer incidence among various tissue types. This correlation, graphically depicted in Fig 1 of [2] for the U.S. and similarly found across the rest of the world [10], is not based on any new assumptions or modeling. It simply points out a previously unrecognized association of data collected by investigators in two diverse fields: epidemiology and developmental biology. Now that this correlation is documented, highly robust, and very statistically significant, it can be considered a reliable experimental result. If a particular theory, particularly one dependent on tenuous assumptions. leads to predictions that are inconsistent with the experimental result, it is more likely that the theory is flawed than the experimental result is flawed.
